# Unfolding the cardioprotective potential of sigma-1 receptor-directed molecular imaging

**DOI:** 10.1007/s12350-022-03077-3

**Published:** 2022-08-04

**Authors:** Takahiro Higuchi, Rudolf A. Werner

**Affiliations:** 1 Department of Nuclear Medicine, University Hospital Würzburg, Oberdürrbacher Str. 6, 97080 Würzburg, Germany; 2 Dentistry and Pharmaceutical Sciences, Okayama University Graduate School of Medicine, Okayama, Japan; 3 Comprehensive Heart Failure Center, University Hospital Würzburg, Würzburg, Germany; 4 The Russell H Morgan Department of Radiology and Radiological Sciences, Division of Nuclear Medicine and Molecular Imaging, Johns Hopkins School of Medicine, Baltimore, MD USA

As a chaperone crucially involved in protein folding,^1^ Sigma-1 receptor (Sig-1R) has been extensively investigated in the context of neurogenerative disorders, such as Parkinson’s or Alzheimer Disease.^1–3^ In recent years, it has also gained interest within the cardiovascular community, as it exerts cardioprotective effects against a.) apoptosis, b.) hypertrophy and c.) maladjusted endoplasmic reticulum stress response.^1^ In this regard, a Sig-1R *in-vivo* molecular imaging approach may not only provide further insights into cardiac ischemia post-myocardial infarction,^4^ but also in the context of hypertrophy-related vascular injury.^1,5^ In the present study, Wakabayashi and coworkers focused on myocardial ischemic burden and the targeting of Sig-1R using the SPECT probe I-125-iodophenyl-piperidino-cyclopentanol (125I-OI5V).^6^ Left coronary occlusion (LCA) was conducted over varying time frames of 10 to 30 min, followed by release of the snare, thereby allowing for reperfusion. 125I-OI5V increased depending on the ischemia time. Second, triple-radiotracer autoradiography including the myocardial perfusion probes 99mTc-MIBI and 201TI revealed that 125I-OI5V uptake ratio was negatively associated with 201TI uptake ratio, supporting the notion that the observed 125I-OI5V accumulation in areas of risk is not only related to the time of LCA, but also depending on reduced 201TI uptake. As such, SIG-1R expression may provide a read-out of the severity of ischemic burden. In addition, 125I-OIV uptake was also mainly located in and adjacent to (CD68-positive) areas of macrophage infiltration.^6^ Although the herein used triple-tracer autoradiography approach is challenging and crucial for understanding the underlying uptake mechanism, the authors may have also provided further evidence on the feasibility of in-vivo imaging, as they have previously demonstrated in an ischemia–reperfusion model limited to 30 min using the identical radiotracer.^4^ Second, the most recent work of Wakabayashi et al. published in this journal would have benefit from a more relevant clinical scenario.^6^ For instance, in transient coronary occlusion models in rats, treatment with the selective Sig-1R agonist, 2-^4-morpholinoethyl^-1-phenylcyclohexane-1-carboxylate hydrochloride (PRE-084) led to improvement in cardiac outcome parameters, such as ejection fraction.^7^ As such, future studies may also consider an image-guided treatment approach.^8^ In this regard, rats could be treated with this Sig-1R antagonist at the time of maximum of target expression as provided by in-vivo 125I-OI5V SPECT and compared to off-peak treated animals, as it has been recently demonstrated for C-X-C motif chemokine receptor-targeted molecular imaging in murine studies.^9^ Such an image-guided treatment would then link the herein observed 125I-OI5V uptake in damaged myocardium to a clinical meaning, in particular as the identical target would be used for both imaging and therapy. Such an assessment of the retention capacities prior to treatment on-set using Sig-1R antagonist would then resemble the widely adopted theranostic concept in nuclear oncology.^10,11^ Nonetheless, the present work by Wakabayashi et al. is of importance,^6^ as it provides the first step towards such an outcome-oriented experimental set-up.

Beyond its use for ischemic burden, other cardiovascular applications would be of even greater interest. As alluded to earlier, the role of Sig-1R in the context of hypertrophy has been already investigated.^5^ In this regard, the vasculo-protective effect of Sig-1R on pressure overload hypertrophy-induced damage in the thoracic aorta has been already demonstrated by using Wistar rats,^5^ i.e., the identical species that had also been used by Wakabyashi et al. in the present study.^6^ In a sophisticated animal model of vascular injury caused by pressure overload, the Sig-1R agonist fluvoxamine led to restored pressure and protected against hypertrophy via upregulation of Sig1-R and stimulation of Sig-1R mediated Akt phosphorylation and endothelial nitric oxide synthase signaling.^5^ Again, using 125I-OI5V in the context of pressure overload and hypertrophy, the Sig1-R retention capacities could be quantified, which may then allow to improve outcome in pressure overload-related vascular damage by initiating fluvoxamine treatment at the maximum of the SPECT signal. This would be even become more relevant if other Sig-1R-directed antihypertensive drugs would be developed.^1^ For instance, mice scheduled for transverse aortic constriction (TAC) could then be used and improvement of contractile function could then be investigated in animals treated at the maximum of 125I-OIV uptake. Of note, such TAC models have just been recently successfully investigated using the most commonly used PET radiotracer 18F-FDG.^12^

Taken together, with its chaperone activity involved in protein folding, a direct *in-vivo* visualization of Sig1-R may be of interest in various clinical scenarios, in particular if Sig-1R drugs providing cardioprotective effects would become available. Until that, the beneficial impact of Sig-1R-targeted molecular imaging in the context of cardiovascular care may remain rather unfolded.
